# Should Bariatric Surgery Candidates Be Screened for Colorectal Cancer? A Systematic Review

**DOI:** 10.3390/jcm15103612

**Published:** 2026-05-08

**Authors:** Basak Kayaalp, Servet Karagul, Cuneyt Kayaalp

**Affiliations:** 1 Department of General Surgery, Faculty of Medicine, Bahcesehir University, Istanbul 34734, Turkey; 2 Department of General Surgery, Faculty of Medicine, Istanbul Atlas University, Istanbul 34408, Turkey

**Keywords:** bariatric surgery, colonoscopy, colorectal cancer, screening, obesity, adenoma

## Abstract

**Introduction:** Obesity and colorectal cancer risk relationship is well-documented and bariatric surgery has become one of the most common surgical operations for losing weight. There is no guideline suggestion for the candidates of bariatric surgery for routine screening of colorectal cancer. The aim of this systematic review is to evaluate the place and necessity of screening colonoscopy before weight loss surgery under the light of published articles. **Methods:** A systematic literature review was conducted in accordance with PRISMA guidelines using electronic databases of PubMed and Google Scholar. The protocol was registered in the International Platform of Registered Systematic Review and Meta-analysis Protocols (INPLASY; registration number: INPLASY202630078). No restrictions were applied regarding the publication dates of the articles. We performed the PRISMA guidelines for systematic review and the PICOS framework was employed. **Results:** A total of 623 patients, 365 women and 258 men, underwent routine colonoscopy before bariatric surgery in three cohort studies. The average age and body mass index of the patients were 47.4 ± 10.4 (range 14–87) and 43.7 ± 7.1 kg/m^2^, respectively. A total of 57 (9.1%) histologically confirmed pathologies were found during colonoscopy, 51 adenomatous polyps (8.1%) and six (1.0%) colorectal cancers. Five of the six CRC patients (83%) were over 45 years of age and three (50%) of them were over 60 years of age. **Conclusions:** The decision to screen bariatric surgery candidates for colorectal cancer should be individualized, based on age (≥45 years), family history, symptomatology, and comorbidities (inflammatory bowel disease). Bariatric surgeons should strongly consider colorectal cancer screening as part of preoperative workup in eligible patients.

## 1. Introduction

Colorectal cancer (CRC) is the third most commonly diagnosed cancer and the second leading cause of cancer-related deaths worldwide [1]. According to GLOBOCAN estimates, more than 1.9 million new cases of CRC and approximately 900,000 CRC-related deaths were recorded in 2022, and these numbers are projected to rise further over the next two decades, primarily driven by population aging, dietary changes, and the global obesity epidemic [2]. Obesity, particularly visceral adiposity, is an established risk factor for CRC and numerous studies have demonstrated a positive association between body mass index and the incidence of CRC [3].

The biological link between obesity and colorectal carcinogenesis is multifactorial. Chronic low-grade inflammation, characterized by elevated circulating levels of interleukin-6 (IL-6) and tumor necrosis factor-alpha (TNF-α), promotes a pro-tumorigenic colonic microenvironment. Insulin resistance and the resulting hyperinsulinemia stimulate the insulin-like growth factor 1 (IGF-1) axis, which favors cell proliferation and inhibits apoptosis in colonocytes. Adipokine dysregulation, with increased leptin and decreased adiponectin, further enhances cellular proliferation and angiogenesis. Finally, obesity-associated alterations of the gut microbiota (dysbiosis) and bile acid metabolism contribute to mucosal injury and may facilitate the adenoma–carcinoma sequence [4,5]. Of particular concern is the rising incidence of early-onset CRC (diagnosed before the age of 50), which has been increasingly attributed, at least in part, to the global rise in childhood and young-adult obesity [6,7].

In parallel, the prevalence of obesity has soared globally, and the number of bariatric procedures has risen accordingly. Bariatric surgery has become a widely accepted and effective treatment for severe obesity, particularly in patients who have not succeeded with conservative weight loss methods [8]. As with any major surgical intervention, preoperative assessment plays a critical role in identifying and mitigating potential risks. In addition to the elevated baseline CRC risk, the bariatric population presents a specific concern after surgery as follows: postoperative anatomical changes (particularly after Roux-en-Y gastric bypass) may delay the recognition of CRC-related symptoms such as iron-deficiency anemia, weight loss, and altered bowel habits, all of which can be misattributed to the surgery itself. Furthermore, endoscopic access to the excluded stomach and proximal small bowel becomes technically demanding after gastric bypass, although the colon remains fully accessible. These considerations strengthen the rationale for performing CRC screening before, rather than after, bariatric surgery in eligible patients [9]. One question that has gained increasing attention is whether candidates for bariatric surgery should be routinely screened for CRC. Given the overlap in risk factors between obesity and CRC, and the potential for missed diagnosis due to anatomical or symptomatic changes post-surgery, this issue warrants careful evaluation. Despite this clinical relevance, current international guidelines for bariatric and metabolic surgery do not provide specific recommendations regarding routine preoperative CRC screening, and practice patterns vary widely between centers and countries [10]. The aim of this systematic review is to evaluate the place and necessity of screening colonoscopy before weight loss surgery under the light of published articles.

## 2. Methods

### 2.1. Protocol and Registration

This systematic review was conducted following the PRISMA guidelines for systematic review [11] and the protocol was registered in the International Platform of Registered Systematic Review and Meta-analysis Protocols (INPLASY; registration number INPLASY202630078). No separate protocol was published. The PICOS framework was employed to structure the research question and ensure the inclusion of suitable studies in the systematic review.

### 2.2. Search Strategies

**Figure 1:**
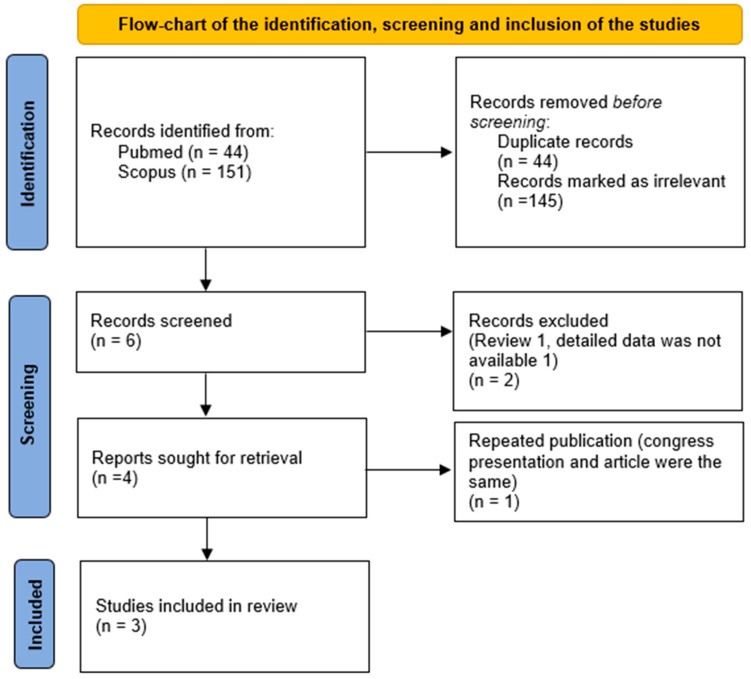
Preferred reporting items for systematic reviews and meta-analysis (PRISMA) flowchart.

Between 1 and 15 March 2026, a systematic literature review was performed by searching the PubMed and Google Scholar databases. No restrictions were applied regarding the publication dates of the articles. The full search strategy for PubMed was as follows: (“bariatric surgery” OR “sleeve gastrectomy” OR “gastric bypass”) AND (“colonoscopy” OR “colorectal cancer” OR “colorectal neoplasia”). For Google Scholar, a similar combination of keywords was used. An updated search using the same strategy was performed on 30 March 2026 to ensure that no relevant articles published during the revision process had been missed; no additional eligible studies were identified. Two authors (CK, BK) independently conducted the search, and after selecting the relevant studies, they examined the references to identify any additional publications that might have been missed (Figure 1). The risk of bias of included cohort studies was assessed using the Newcastle–Ottawa Scale ((NOS; Wells GA, Shea B, O’Connell D, et al., Ottawa Hospital Research Institute, Ottawa, ON, Canada). Disagreements were settled via discussions with a different third author (SK) before an agreement was reached. Studies were grouped based on outcome (adenoma and CRC prevalence). The primary effect measure was prevalence (%) of colorectal neoplasia (adenoma and CRC). Results were presented using tables and forest plots.

P (Participants): All bariatric surgery candidates in the study groups, regardless of age and gender, were included in the systematic review. There were no limitations or selection according to the surgical types.

I (Intervention): Studies in which colonoscopy screenings were done before bariatric surgeries were included. Colonoscopy following surgery or other preoperative screening methods such as fecal occult blood test or cDNA studies were excluded.

C (Comparison): There were no comparison groups. We analyzed the prevalence of colorectal tumor-related pathologies that were diagnosed during preoperative colonoscopy screening.

O (Outcome): Only the prevalence of CRC and adenomas were calculated. Other pathologies apart from these were recorded but not accepted for analyses.

S (Study Design): Cohort studies were included. Case reports and other materials not including data were not accepted for the systematic review.

### 2.3. Statistics

A computer program including spreadsheet was used for records (Microsoft Excel, Microsoft 365, Microsoft Corporation, Redmond, WA, USA). Data were tabulated in tables, and column sums were created with the numbers. Basic calculation methods were used for statistical analysis of both dichotomous and continuous variables. MedCalc statistical program was used for cumulative meta-analysis proportion calculation (MedCalc^®^ Statistical Software (version 22, MedCalc Software Ltd., Ostend, Belgium). When studies reported the median and range, we estimated the mean and standard deviation using the method described by Hozo et al. [12]. Publication bias was assessed visually using funnel plots (generated in MedCalc^®^ Statistical Software, version 22, MedCalc Software Ltd., Ostend, Belgium). The certainty of evidence was not formally assessed.

## 3. Results

There were 45 and 151 reports in PubMed and Google Scholar databases, respectively. Studies were excluded due to lack of preoperative colonoscopy data, non-cohort design, or insufficient outcome reporting. Following the selection, only three studies were found as eligible for the review [13,14,15] (Figure 1).

The first study on this subject was published in 2014 as a joint study of the United States and the United Arab Emirates. The second study came from Türkiye in 2019. The last research was from Japan in 2024. The included studies were assessed as moderate quality according to the NOS. A total of 623 patients, 365 women and 258 men, underwent routine colonoscopy before bariatric surgery. The average age and body mass index of the patients were 47.4 ± 10.4 (range 14–87) and 43.7 ± 7.1 kg/m^2^, respectively (Table 1).

**Figure 2:**
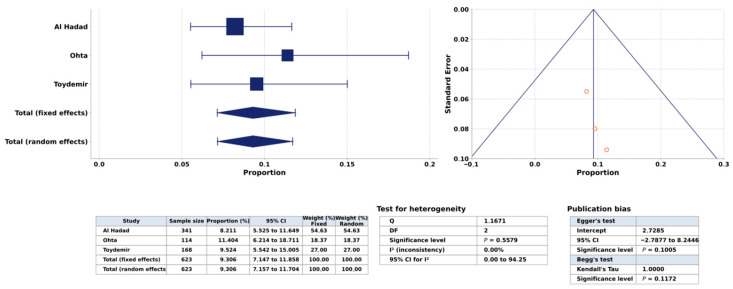
Forest plot showing pooled prevalence of colorectal neoplasia and funnel plot assessing publication bias in bariatric surgery candidates undergoing preoperative colonoscopy [13,14,15].

A total of 57 (9.1%) histologically confirmed pathologies were found during colonoscopy, 51 adenomatous polyps (8.1%) and six (1.0%) CRC (Figure 2).

Five of the six CRC patients (83%) were over 45 years of age and three (50%) of them were over 60 years of age. Three of the patients were female and three were male. All men were over 60 years of age. The women were between the ages of 40 and 57. The first study by Al Haddad et al. (2014) highlighted the alarming risk for the development of colonic polyps and CRC at bariatric surgery candidates and their study was a plea for international bariatric societies to establish clear guidelines for screening colonoscopy during the workup of morbidly obese patients before they undergo bariatric surgery [13]. Toydemir and co-workers (2019) warranted routine screening colonoscopy in the 40–49-year-old morbidly obese and/or metabolically symptomatic patient population with average-risk and in aged > 50, CRC screening by colonoscopy must be enforced [14]. The last study by Ohta et al. (2024) concluded that older age and male sex may be risk factors for CRC in obese Japanese candidates for laparoscopic bariatric/metabolic surgery, and preoperative colonoscopy should be considered for these high-risk patients [15]. Due to the limited number of studies, subgroup and sensitivity analyses were not performed and the certainty of evidence is limited. Funnel plot analysis suggested no significant publication bias.

## 4. Discussion

Obesity and CRC risk relationship is well-documented and its mechanisms include: chronic inflammation (elevated cytokines such as IL-6 and TNF-α), insulin resistance and hyperinsulinemia (promoting tumor growth via IGF-1), altered gut microbiota (dysbiosis contributing to carcinogenesis) and increased adipokines (leptin’s proliferative effects vs. adiponectin’s protective role) [4]. A meta-analysis by Ma et al. found that obese individuals have a 20–30% higher risk of CRC than those with normal BMI. Given this elevated risk, screening bariatric surgery candidates may be warranted [3]. The U.S. Preventive Services Task Force (USPSTF) recommends CRC screening starting at age 45 for average-risk individuals [16]. However, bariatric surgery patients often have additional risk factors (e.g., metabolic syndrome, chronic inflammation), suggesting that earlier or more intensive screening may be beneficial.

The adenoma detection rate (ADR), which represents the proportion of colonoscopies in which at least one adenoma is detected, serves as a performance indicator for colonoscopists. Studies have shown that a 1% increase in the ADR is associated with a 3% reduction in CRC incidence and a 5% reduction in CRC-related mortality [17]. It is illuminating to compare the findings from our current study with the prevalence rates reported in screening cohorts. In prospective multicenter studies, the ADR has been reported as 15–38%. The CRC detection rate, however, has been reported to range from 0.7% to 3.9% [18,19,20,21,22]. In our study, the adenomatous polyp rate was 8.1%, and the CRC rate was 1%. We can attribute the lower ADR in our study compared to the aforementioned studies to the lower mean age (e.g., the Japanese cohort had a mean age of 44 years). In a systematic review by Fernandes et al., a total of 3,644,561 patients underwent colonoscopy. None of the included studies reported a mean age under 45 years, and obesity was not described or available in all studies [23]. From this perspective, we believe our data will contribute to new colonoscopy screening studies. Furthermore, the relatively higher prevalence of CRC despite the younger age distribution is consistent with the increased CRC risk associated with obesity, strengthening the rationale for targeted preoperative screening in this population.

Beyond body mass index, the duration of life spent obese is increasingly recognized as an independent risk factor for colorectal cancer. The “pack-years” concept, which is used for tobacco-related diseases, has been applied similarly to obesity as “obese-years” [24,25]. The long-term presence of visceral obesity creates a carcinogenic microenvironment characterized by chronic low-grade inflammation, hyperinsulinemia and adipokine imbalance. Patients seeking bariatric surgery usually have a long-standing history of obesity. However, as the duration of obesity was not reported in the studies included in our review, our current evidence is limited, and this presents an opportunity for future prospective research.

The elevated baseline CRC risk suggests that bariatric surgery candidates may already fall into a higher-risk category for CRC and thus benefit from screening. The preoperative period represents a valuable window for health screening. Identifying and addressing CRC before surgery can not only improve patient outcomes but also alter surgical plans if necessary. Although there are no universally mandated protocols for CRC screening specifically in bariatric surgery patients, general guidelines support CRC screening in adults aged 45–75, particularly those with additional risk factors [16]. Implementing CRC screening in bariatric patients who meet age- or risk-based criteria aligns with these guidelines and may be cost-effective when weighed against the potential costs of advanced-stage cancer treatment.

There can be some arguments against routine CRC screening for all bariatric surgery candidates. Despite the theoretical and epidemiological rationale, there is limited direct evidence that preoperative CRC screening in bariatric candidates leads to improved long-term outcomes. Routine preoperative colonoscopy or other CRC screening procedures can add to healthcare costs, scheduling delays, and patient anxiety. In younger patients or those with no family history or symptoms suggestive of CRC, the benefits of screening may not outweigh the burdens. Some data suggest that bariatric surgery may actually reduce long-term CRC risk due to weight loss and metabolic improvements [26]. If confirmed, this would challenge the urgency of screening preoperatively, especially in younger individuals without other risk factors. Bariatric surgery has become one of the most common general surgical operations today. Although a significant part of the patients who have undergone weight loss surgery are at a younger age, bariatric surgery is performed on middle-aged and elderly patients day by day. While the implementation of weight loss surgeries at an advanced age was very discouraged in the past, today the guidelines have removed the upper age limit [10]. For this reason, surgeons are now performing weight loss operations on much older patients. It should be kept in mind before surgery that an elderly patient who will undergo obesity surgery may have synchronous CRC. Currently, CRC screenings are recommended from the age of 45, with or without obesity, and it is recommended that patients over the age of 45 who will undergo bariatric surgery should be screened by colonoscopy.

**Figure 3:**
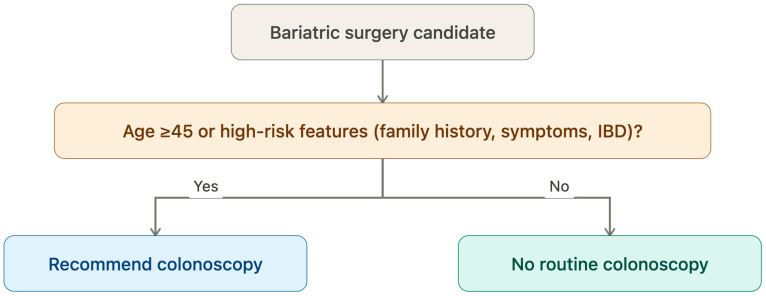
Proposed decisional algorithm for preoperative colonoscopy in bariatric surgery candidates.

Building on the available evidence and the points raised above, we propose a simple, clinically applicable decisional algorithm for preoperative colonoscopy in bariatric surgery candidates (Figure 3). The algorithm stratifies candidates by age (≥45 vs. <45 years) and by the presence of additional risk factors, defined as a positive family history of CRC, gastrointestinal symptoms suggestive of colonic pathology (rectal bleeding, unexplained iron-deficiency anemia, and change in bowel habits) or inflammatory bowel disease. Preoperative colonoscopy is strongly recommended for all candidates aged ≥ 45 years and for younger candidates with one or more risk factors. Younger asymptomatic candidates without any risk factor may be managed according to standard population-based screening recommendations. The algorithm is intended as a pragmatic decision-support tool rather than a rigid rule, and its diagnostic yield and cost-effectiveness should be validated in prospective studies.

In addition to increased body mass index in obese individuals, visceral adipose tissue exhibits the characteristics of an organ with metabolic activity, leading to insulin resistance and hyperinsulinemia, thereby triggering carcinogenesis [27]. The resulting metabolic environment accelerates cell growth, and apoptosis is inhibited by hormonal factors, increasing the risk of colorectal neoplasia. Molla and colleagues noted that the risk of developing conventional adenomas was significantly increased in both metabolically healthy obesity and metabolically unhealthy obesity groups and that this increase was more pronounced in male participants [28]. Body fat distribution may guide us toward identifying a group of patients for whom we should prioritize colonoscopic examination based on phenotype, but more studies are needed in this area. Our analysis does not have sufficient data to provide a definitive view on this matter or to determine an approach to patients based on body type. Although the molecular mechanism underlying the association between obesity and colorectal cancer has not been fully elucidated, adipocytes are activated by the secretion of hormones such as leptin and adiponectin, which may trigger the development of colorectal neoplasia in obese individuals without metabolic disease by inducing obesity-associated chronic inflammation. A decrease in skeletal muscle mass may increase the risk of colorectal adenoma development [27,29]. Li et al. noted that a low skeletal muscle mass index is associated with the presence of inflammatory and serrated polyps in men. The current evidence is unclear, and further investigation is needed into the relationship between skeletal muscle mass and the development of colorectal adenoma. Insulin resistance plays a role in colorectal carcinogenesis through the transformation of colon mucosal cells. Sarcopenia, however, reduces the effect of skeletal muscle in preventing insulin resistance. Sarcopenia and visceral obesity influence common pathophysiological mechanisms such as insulin resistance, oxidative stress, inflammatory cytokines, and hormonal changes [30]. Phenotype characteristics and skeletal muscle parameters were not included in the present study as a limitation. If more comprehensive studies are conducted, examining these factors alongside diet, physical activity characteristics, and family history—and conducting prospective studies—could help develop more robust algorithms for patient selection in preoperative colonoscopy before bariatric surgery.

If a patient who has undergone obesity surgery over the age of 45 has an overlooked CRC, this can also create problems from a medical point of view. When CRC symptoms appear shortly after surgery, patients may complain because the cancer was not detected before or during surgery and because of the delay in diagnosis. For this reason, bariatric surgery candidate patients over the age of 45 or young patients with high-risk (family history, symptomatic or inflammatory bowel disease) should be recommended to have a colonoscopy. Even if patients do not do this, it is important for the bariatric surgeon to recommend it and record this recommendation in writing in terms of the safety of the patient and himself. This systematic review does not bring any innovations to the known CRC screening recommendations. Actually, these recommendations are valid for people who will undergo a laparoscopic cholecystectomy for gallstones as well. The aim of this study is to remind bariatric surgeons of the risk of CRC in their patients, to recommend colonoscopy when indicated, and to ensure that they take legal action.

This study has several limitations. Firstly, the number of studies that could be included was limited, and little evidence was found regarding the screening of colon and rectal cancers before bariatric surgery. The designs of the included studies also vary. They differ in terms of patient demographics, risk groups and geographic regions, resulting in clinical and methodological heterogeneity. The sample size and number of cases identified may reduce the statistical sensitivity of colorectal screening. In addition, none of the included studies reported obesity duration, family history details in a structured manner, or the prevalence of metabolic syndrome components, which prevented us from analyzing these variables in relation to colorectal neoplasia. Furthermore, due to the small number of studies, publication bias cannot be ruled out in the evaluation. Therefore, caution is needed when interpreting the findings, and large-scale randomized studies must be designed.

## 5. Conclusions

An analysis of three eligible cohorts (*n* = 623) revealed that preoperative colonoscopy identified colorectal pathology in 9.1% of bariatric surgery candidates. Of these, 8.1% had adenomas and 1% had colorectal cancer. Most cases of colorectal cancer were observed in patients aged 45 and over. While colonoscopy is not required for all bariatric surgery candidates, targeted preoperative screening is recommended for patients aged 45 and over, as well as for those with a family history of the disease, gastrointestinal symptoms or inflammatory bowel disease. Bariatric surgeons should adopt this risk-stratified approach, document the patient’s decision and contribute to prospective studies that will inform the development of guidelines. Detecting a treatable malignancy prior to surgery offers a realistic opportunity to protect the health of patients and shield the surgical team from potential legal consequences.

## Figures and Tables

**Table 1:** Characteristics of included studies.

Author	Year	Country	No	Age	Female	Male	BMI kg/m^2^	Adenoma	CRC	Total
Al Hadad [13]	2014	USA and UAE	341	45 (14–87) 47.8 ± 12.2	199	142	NA	23	5	28
Toydemir [14]	2019	Turkey	168	48.9 ± 5.9 (40–65)	94	74	43.7 ± 6.6 (31.1–70.5)	15	1	16
Ohta [15]	2024	Japan	114	23–65 (44.2 ± 9.0)	72	42	43.8 ± 7.9	13	0	13

## Data Availability

All data generated or analyzed during this study are included in this published article and its Supplementary Materials.

## Supplementary Materials

The following supporting information can be downloaded at https://www.mdpi.com/article/10.3390/jcm15103612/s1, Table S1: PRISMA 2020 checklist [11].

## Abbreviations

BMI	Body Mass Index
CRC	Colorectal Cancer
PRISMA	Preferred Reporting Items for Systematic Reviews and Meta-Analyses
INPLASY	International Platform of Registered Systematic Review and Meta-analysis Protocols
PICOS	Population, Intervention, Comparison, Outcome, Study Design
CI	Confidence Interval
IGF-1	Insulin-like Growth Factor 1
IL-6	Interleukin-6
TNF-α	Tumor Necrosis Factor-alpha
USPSTF	United States Preventive Services Task Force
ADR	Adenoma Detection Rate
